# Relationship between nanotopographical alignment and stem cell fate with live imaging and shape analysis

**DOI:** 10.1038/srep37909

**Published:** 2016-12-02

**Authors:** Peter Newman, Jorge Luis Galenano-Niño, Pamela Graney, Joselito M. Razal, Andrew I. Minett, João Ribas, Raquel Ovalle-Robles, Maté Biro, Hala Zreiqat

**Affiliations:** 1 Biomaterials and Tissue Engineering Research Unit, School of Aeronautical Mechanical and Mechatronics Engineering, University of Sydney, Sydney, NSW, 2006, Australia; 2 EMBL Australia node in Single Molecule Science, School of Medical Sciences, The University of New South Wales, Sydney, Australia; 3 Department of Biomedical Engineering, School of Biomedical Engineering, Science and Health Systems, Drexel University, Philadelphia, PA 19104, USA; 4 Institute for Frontier Materials, Deakin University, Geelong, Victoria, 3216, Australia; 5 Laboratory for Sustainable Technology, Department of Chemical and Biomolecular Engineering, University of Sydney, NSW, 2006, Australia; 6 Australian Institute for Nanoscale Science and Technology, University of Sydney, NSW, 2006, Australia; 7 Biomaterials Innovation Research Center, Department of Medicine, Brigham and Women’s Hospital, Harvard Medical School, Cambridge, Massachusetts 02139, USA; 8 Nano-Science & Technology Center, LINTEC of America Inc., Richardson, Texas 75081, USA; 9 Sydney Medical School, The University of Sydney, NSW, 2006, Australia

## Abstract

The topography of a biomaterial regulates cellular interactions and determine stem cell fate. A complete understanding of how topographical properties affect cell behavior will allow the rational design of material surfaces that elicit specified biological functions once placed in the body. To this end, we fabricate substrates with aligned or randomly organized fibrous nanostructured topographies. Culturing adipose-derived stem cells (ASCs), we explore the dynamic relationship between the alignment of topography, cell shape and cell differentiation to osteogenic and myogenic lineages. We show aligned topographies differentiate cells towards a satellite cell muscle progenitor state - a distinct cell myogenic lineage responsible for postnatal growth and repair of muscle. We analyze cell shape between the different topographies, using fluorescent time-lapse imaging over 21 days. In contrast to previous work, this allows the direct measurement of cell shape at a given time rather than defining the morphology of the underlying topography and neglecting cell shape. We report quantitative metrics of the time-based morphological behaviors of cell shape in response to differing topographies. This analysis offers insights into the relationship between topography, cell shape and cell differentiation. Cells differentiating towards a myogenic fate on aligned topographies adopt a characteristic elongated shape as well as the alignment of cells.

Tissue engineering aims to return healthy function to damaged tissue. A common strategy uses three dimensional synthetic scaffolds that return tissue function by supporting the regrowth of healthy cells. Within a scaffold environment, cell behavior is regulated by a complex integration of biochemical, mechanical and architectural cues from the scaffold. Understanding the effect of these biophysicochemical cues on cell behavior would pave the way for fabricating tailored scaffold structures that elicit a specified function once placed in the body.

The mechanical and architectural properties of a scaffold were traditionally considered to provide permissive conditions under which biochemical stimuli controlled cell behavior^1^. Biochemical cues, including growth factors, were considered paramount in promoting cell proliferation and regulating stem cell fate during tissue regrowth. Accumulating evidence demonstrates that the physical properties of a cellular environment play a role in controlling cell fate. Researchers are exploring the different ways physical environments can alter mechanotransductive signaling and downstream cell behaviors. In 2006, seminal work by Engler *et al*. showed elastic properties of the extracellular environment influence cell fate of human mesenchymal stem cells (hMSCs)^2^. They demonstrated soft substrates induced neurogenesis, while stiffer substrates induced myogenesis, and comparatively rigid substrates promoted osteogenesis; a result corresponding to the native tissue elasticity of the induced phenotype. These findings suggest that differences in substrate stiffness provide differences in the forces transmitted and sensed by cells in that environment. Through such mechanotransductive processes cells convert physical stimuli into biochemical signals thereby regulating cell proliferation and differentiation behaviors.

This has initiated important research studies towards exploring the different ways physical environments can alter mechanotransductive signaling and downstream cell behaviors. In addition to differences in stiffness, the architecture of the extracellular environment influences cell behaviors. For example, during embryogenesis, cells at the periphery of a developing organism experience different architectural cues to those on the inside. This spatial polarity alters mechanotransductive pathways and leads to segmentation and the eventual formation of defined tissue architectures^3^ with Par (partitioning defective) complex (composed of Par3, Par6, and aPKC) and associated Rho GTPase signaling, implicated as regulators of cell polarity during such processes^4^. Similar effects have been created by confining the area to which a cell can attach. Cells confined to areas with relatively low compactness (the ratio of a shape’s circumference divided by its area), such as a circle, results in lower intracellular forces, compared to areas with high compactness, including long and thin rectangles, in which cells experience higher intracellular forces^5^. Furthermore, populations of cells confined to areas of high compactness experience large intracellular forces that direct differentiation towards tissue types that physiologically experience high forces, as is the case in cartilage and bone. Conversely, cells confined to areas of low compactness will experience less force and differentiate towards tissues that experience low physiological forces such as adipose tissue^6^.

Changing the topography of the surface to which cells attach is another way in which cell mechanotransduction is modulated. The influence of differing topographies reflects that of confining cell adhesion^7^. Topographical features of varying size, shape and spacing can determine cell attachment, integrin clustering, cytoskeletal structure, cell shape and cell polarity altering their mechanotransductive signaling and in turn downstream behaviors including differentiation^1^,^4^,^8^. For example, topographies with small round features can restrict cell spreading to small areas and rounded shapes with lower intracellular stress thereby promoting adipogenic behaviors, whereas those with large elongated features can promote cell spreading and large cell areas with elongated cell shapes and higher intracellular stress that favor osteogenic behavior^6^,^9^. Such surfaces have been used to specify differentiation for a range of tissue types including osteogenic^9^,^10^,^11^,^12^, tenogenic^13^, chondrogenic^14^, myogenic^15^,^16^,^17^,^18^, adipogenic^19^ and neurogenic tissues^20^,^21^. This is appealing in the field of tissue engineering where topographical surface modifications offer a simple and cost-effective alternative to traditional differentiation techniques that would otherwise require growth factors or other biochemicals that are relatively unstable and expensive.

One such topographic modification is the use of aligned nanofeatures. Numerous previous reports demonstrate alignment of extracellular nanofeatures corresponds with alignment of cell cultured over such environments as well as elongation of cell shape^17^,^18^,^22^,^23^,^24^,^25^. Similar to confinement effects, topographically induced aligned and elongated cells experience altered mechanotransductive signaling, including integrin clustering, cytoskeletal organization and altered intracellular forces^8^. It is suggested that aligned topographies promote cell spreading and elongation through contact guidance effects – where continuous aligned features passively bias anisotropic cell spreading^9^,^26^. Stem cells spread over environments with aligned anisotropies favor osteogenic and myogenic lineages^23^,^24^,^27^.

Despite the potential of surface topography to guide cell differentiation, the relationship between topography and stem cell fate is still poorly understood. This includes the complex force dependent mechanotransductive pathways that link a cell’s environment to its transcriptional machinery, as well as the environmental cues that initiate these forces. A deeper understanding of the relationship between these properties will allow for the rational design of material surfaces capable of directing cell shape and differentiation. To this end we explore the dynamic relationship between alignment of topography, cell shape and cell differentiation. Primary human adipose derived stem cells (ASCs) were cultured on glass surfaces coated with nanotopographies of random and aligned nanostructures. The osteogenic and myogenic differentiation of ASCs cultured on the different topographies were determined using colorimetric assays and quantitative real-time reverse transcription polymerase chain reaction (qRT-PCR). To understand how different topography alters cell shape, we use fluorescent time-lapse imaging over 21 days, subsequently comparing shape metrics between the different topographies. While previous reports have quantified cell shape at discrete time points, ours is the first to quantitatively report cell morphologies in response to differing topographies across long time periods. This analysis offers insights into the relationship between topography, cell shape and cell differentiation. We show that alignment of topography significantly increases cell area and elongation. This shape reflects the underlying aligned topography which may promote directed formation through contact guidance effects. Comparatively, random topographies support cells with a small area and relatively round shapes. Random topographies lack consistent features that would otherwise promote directed formation through contact guidance effects. Contrary to previous reports, this study demonstrates that an aligned topography does not upregulate osteogenesis^27^,^28^. Alternatively, we demonstrate that relative to random topographies, aligned topography shows high expression of the early stage myogenic differentiation factors PAX7 and MYF5 – suggesting differentiation towards a satellite cell muscle progenitor state - a distinct myogenic cell lineage responsible for postnatal growth and repair of muscle.

## Results

**Figure 1:**
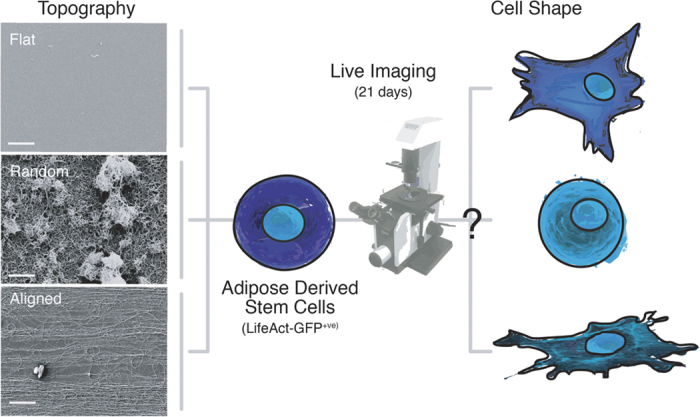
Scheme of experimentation – different topographies were fabricated and seeded with adipose-derived stem cells. The influence of substrate topography, on cell fate is examined with live imaging of cells over 21 days and cell shape and differentiation were measured. Scanning electron micrographs of uncoated glass coverslip or flat topography, random topography and aligned topography are shown at the left of Fig. 1. The alignment of the nanotopography in aligned substrates is horizontal across the page – scale bars 1 μm.

Random and aligned topographies were fabricated by coating glass coverslips with carbon nanotubes (CNTs) from an aligned drawable forest^29^ (Figure S1), where uncoated glass coverslips served as the flat topography. The different topographies of the substrates are shown in Fig. 1. Details showing the fabrication and characterization of the substrates are included in supplementary information and shown in Figures S1–4.

### Effect of osteogenic media and nanotopography on ASCs differentiation

**Figure 2:**
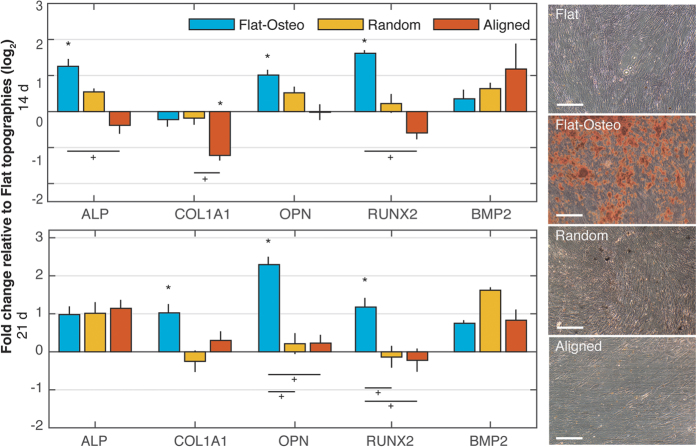
Osteogenic characterization of ASCs grown over flat, flat-osteo, random and aligned topographies. qRT-PCR results for osteogenic gene expression including alkaline phosphatase (*ALP*), collagen, type 1, alpha 1 (*COL1A1*), osteopontin (*OPN*) and runt related transcription factor 2 (*RUNX2*) and bone morphogenetic protein 2 (BMP2) was calculated using the ∆∆CT method and is shown as the log_2_ fold change relative to flat topographies. Osteogenic gene expression is upregulated comparing flat and flat-osteo conditions. Otherwise no significant or consistent trend is observed between topographies. (*shows statistical significance to flat topographies, P < 0.05; + shows statistical significance between topographies, P < 0.05; unpaired two sample Student’s t-test comparing ∆CT values is used throughout). Micrographs at right show Alizarin Red staining of ASCs grown over flat, flat-osteo, random and aligned topographies. Red stained calcific deposits are prominent for flat-osteo topographies (Scale bars 100 μm).

Osteogenic differentiation of ASCs cultured on the different topographies using qRT-PCR (Fig. 2). As a positive control for osteogenesis, ASCs were cultured on flat topography glass coverslips in osteogenic media – referred to as ‘flat-osteo’ topographies. Relative to flat topographies, flat-osteo conditions show significant upregulation of mRNA expression levels for osteopontin (*OPN*) and runt related transcription factor 2 (*RUNX2*) at both 14 and 21 days as well as alkaline phosphatase (*ALP*) at 14 days. Collagen, type 1, alpha 1 (*COL1A1*) was upregulated at day 21. In contrast, osteogenic gene expression is not altered when ASCs were cultured on flat, random and aligned topographies at either 14 or 21 days.

We then compared the effects of nanotopography on mineralization of the cells with alizarin red staining following 21-day of culture (Fig. 2). Relative to all other topographies ASCs cultured on flat-osteo show prominent red stained calcific deposits. In agreement with the qRT-PCR results, neither flat, random or aligned topographies alter mineral deposition when compared to flat-osteo conditions at day 21.

**Figure 3:**
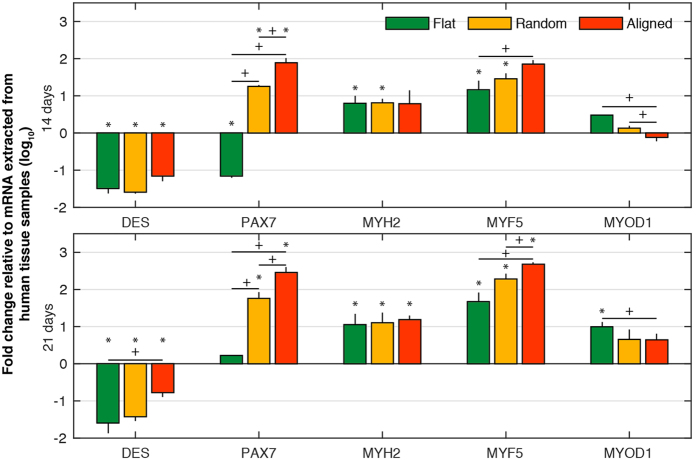
Myogenic expression over different topographies – qRT-PCR results for ASCs grown over flat, random and aligned topographies. Relative expression of Desmin (*DES*), paired box 7 (*PAX7*), myosin heavy chain 2 (*MYH2*), myogenic factor 5 (*MYF5*) and myogenic differentiation 1 (*MYOD1*) was calculated using the ∆∆CT method and is shown as the log_10_ fold change relative to flat topographies. Myogenic marker *PAX7* is upregulated at 14 and 21 days from both random and aligned topographies compared to flat topographies. Aligned topography shows further upregulation of *MYF5* at day 14 and both *DES* and *MYF5* at day 21. *MYOD1* is significantly down regulated at 14 and 21 days for aligned topographies. (* shows statistical significance between control group and topographies, P < 0.05; + shows statistical significance between topographies, P < 0.05; unpaired two sample Student’s t-test comparing ∆CT values is used throughout).

Given the well-established potential for surface topography to direct stem cell fate and the morphological similarity between cells cultured over aligned topographies and those of myogenic lineage, we hypothesized that differentiation steers towards a myogenic fate – a result that has been demonstrated for aligned topographies and mesenchymal stem cells (MSCs)^9^,^10^,^25^,^26^,^27^. We extended qRT-PCR analysis to examine the myogenic markers Desmin (*DES*), paired box 7 (*PAX7*), myosin heavy chain 2 (*MYH2*), myogenic factor 5 (*MYF5*) and myogenic differentiation 1 (*MYOD1*) as reported elsewhere (Fig. 3)^30^,^31^. Relative to other topographies, aligned topographies showed significant downregulation of *MYOD1* and upregulation of markers *PAX7* and *MYF5* at 14 and 21 days. Expression of *PAX7* was significantly upregulated for random topographies at 14 and 21 days.

### Effect of nano-topographical cues on cell shape

**Figure 4:**
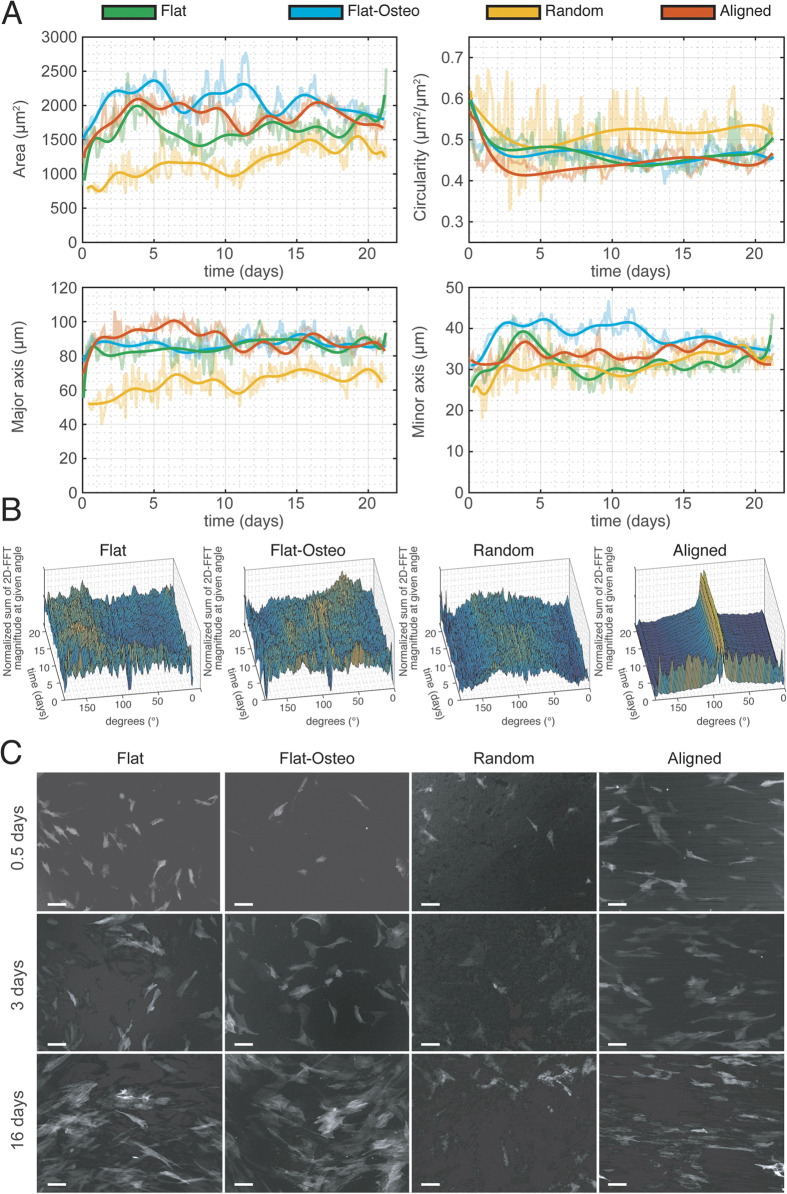
Cell shape analysis between different topographies. Panel A shows the cell shape metrics for 21 d culture of ASCs over flat, flat-osteo, random and aligned topographies. The shape metrics area, circularity, major axis length and minor axis length are taken from image analysis completed with CellProfiler. Panel B shows the relative magnitude of features (z axis) at a given angle (x axis) over the 21 d (y axis) culture period is shown. The relative magnitude of different angles shows strong and consistent alignment for aligned topographies. Panel C shows representative images of flat, flat-osteo, random and aligned topographies at 0.5, 3 and 16 day time points (scale bars 100 μm).

We directly measured cell shape over 21 days using fluorescent time-lapse imaging (Incucyte ZOOM). We used this analysis to examine the effect that topographical cues impart to the shape of ASCs. We examined the cell shape metrics of cell area, circularity, major axis and minor axis. We defined circularity of a cell as the scaled ratio of it’s area and perimeter – equal to 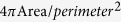; equal to 1 for a perfectly circular object and decreases to 0 for shapes with an increasing perimeter for a given area. The major and minor axis of the cell are respectively defined as the longest and shortest axis of the smallest ellipse that completely encloses a cell, representing therefore a cell’s approximate length and width. These metrics are known to relate to cell morphology of the myogenic and osteogenic phenotypes – myogenic cells with large major axes and a relatively small minor axis^5^,^6^,^32^, and osteogenic cells with large areas and major axis^5^. These were calculated and organized into time series plots in Fig. 4A–B (processing of fluorescent time-lapse images completed in CellProfiler software^33^, details in SI).

There are general trends seen in cell shape over the 21 day culture period. There is an increase in cell area and major/minor axis for all topographies from 0 to 1 day. This attachment behavior reflects the time series plot of cell circularity in Fig. 4A. Cells are initially circular but quickly spread and consequently lose their circularity; thereafter, circularity remains constant. The cell shape of ASCs cultured over random topographies is the most dissimilar to other topographies. The ratio of the mean area, circularity and major/minor axis value between any two topographies is furthest from unity when comparing with random topographies.

Given that all cells initially displayed similar non adherent circular shapes, we examined the time taken for cell shape to become dissimilar between different topographies. Table 1 summarizes this data (see Supplementary information for additional information). Cell shape remained most similar between aligned and flat topographies with similar (F-test, P > 0.05) area, circularity, major axis and minor axis for the first 1.5, 504, 1.25 and 0.5 hours respectively. Despite sharing flat topography, cell area on flat and flat-osteo conditions diverged quicker than flat and aligned topographies. This indicates that the effect of biochemical stimulus dominates those of topography when influencing cell shape.

The underlying topography dictates the area of ASCs. The time taken for cell area to vary between all pairs of given topographies is 1.5 hours (Table 1). Comparing conditions with similar media, cells cultured on aligned topographies had the largest area. Flat topographies supported the second smallest cell area and random topography supported the smallest cell area over the course of the experiment (see discussion on effect of contact guidance below).

Osteogenic media altered cell shape. Comparing cell area between flat and flat-osteo topographies demonstrates that osteogenic differentiation increases cell area. The addition of osteogenic media to flat topographies regulates cells shape to support larger areas. Comparing the major axis length of flat-osteo and flat topographies reveals these metrics are comparable from 4.5 through to 21 days (Figure S10). This suggests that ASCs under osteogenic differentiation increase cell area by spreading in all directions and not along one axis – flat-osteo topographies having both the largest average area and the largest average minor axis.

Further, cells cultured on random topography were the most circular when compared to flat, aligned and random topographies. Cells on flat and flat-osteo topographies have comparable circularity throughout (F-test – see Figure S10 for more information). Cells on aligned topographies generally have the lowest circularity during the 21 days of culture, this reflects the features of the underlying topography and their resulting elongated shape with large major axis and small minor axis.

ASCs cultured on aligned topographies have an elongated shape supporting the largest mean major axis length. This shape reflects features of the underlying aligned topography which may promote directed elongation through contact guidance effects^26^. Additional support for this behavior is observed through the inability of random topographies to support large area or major axis lengths. Random topographies lack organized features that promote directed elongation through contact guidance effects.

### Effect of nano-topographical cues on cell alignment

Cell alignment was quantified by summing the magnitude of the two dimensional fast Fourier transforms (2D-FFTs) of fluorescent time-lapse images between angles 0 to 180°. This method is advantageous as it provides orientation distributions for images of overlapping features. Further, its speed and robust nature make it the preferred calculation method for quantification of alignment distributions in large data sets. An image with a 2D-FFT showing a high magnitude at a given angle indicates that the image contains pixels of similar intensity aligned at that particular angle. As shown in Fig. 4B cells initially had no preference in their orientation across all topographies. Specifically, at day 0 the relative magnitude of any given angle is comparable across all topographies. This reflects the absence of elongation in a specific direction and thus circularity metrics at day 0. Following from day 0 through to 21 days, a consistent and high degree of alignment is evident for aligned topography (Fig. 4B) as reflected in the prominent peak in the normalized sum of 2D-FFT magnitude at ~90° for all time points. Comparing bright field and fluorescent images confirms the alignment of the underlying topography is at ~90°. A prominent peak is not present for flat, flat-osteo and random topographies at any given time point. This indicates that there is no consistent global alignment of cells when cultured over these topographies.

## Discussion/Conclusion

The morphology of different cell phenotypes supports their function. Fat cells are round to maximize the fat they can store, and muscle cells are elongated and aligned such that actomyosin fibers align and contract in unison – that is, cell function follows its form, and cell form can be altered to regulate its function. Changes to the form of the extracellular environment regulate cell differentiation by influencing cell shape as well as mimicking the organization and anisotropies of ECM structures. Such organization of cells is ubiquitous in nature, particularly in muscle tissue where organization is critical to function. Without the organization of actomyosin in aligned filaments, and alignment of cells along fibres, the actions of cell-scale contractions would not sum and macroscale function would be lost. Additional examples include embryogenesis in which environmental anisotropies define morphogenetic processes such as the formation of the mesendoderm^34^. It is believed that such anisotropies are reflected through a cells shape and the forces on the cell’s nucleus which then regulate transcription^16^,^35^. For example, stem cell differentiation has been linked to the shape of a cellular nucleus and nuclear lamin-A proteins, which are regulated in response to changing physical environments such as substrate elasticity^36^.

We examined the relationship between the cellular physical environment and cell shape using fluorescent time-lapse microscopy. Importantly, we directly measure cell shape over the different topographies. This is in contrast to previous work that instead defines the morphology of the topography to which cells attach neglecting the shape of the cells following attachment. This includes extensive quantitative reporting of the time-based morphological behavior of ASCs attaching to different topographies. While previous reports have quantified cell shape at discrete time points, ours is the first to report quantitative time based behaviors of stem cell morphology in response to differing topographies across long time periods. This reporting gives insights into the effects that underlying topographies have on cell shape. Our report shows that nanotopographical alignment is able to induce alignment of ASCs when cultured on such a topography. With these methods we have determined the dynamic differences between differing topographies. We further demonstrated that topography alters other cell shape parameters including cell area, circularity and length of the major/minor axis. Specifically, cells cultured on aligned topographies have relatively large areas and major axis (Fig. 4A). This shape was reflected in the features of the underlying aligned topography which may promote directed formation through contact guidance effects^26^. Comparatively, random topographies support cells with a small area. Random topographies lack consistent features that would otherwise promote directed formation through contact guidance effects. The observed changes to cell shape correspond with altered osteogenic and myogenic expression. Myogenesis increased over aligned topographies relative to flat and random topographies. These increases reflect previous reports that suggest alignment and cell elongation positively influence myogenesis^16^,^18^,^23^,^24^,^37^,^38^,^39^. Interestingly, upregulation of the myogenic progenitor marker PAX7 is also seen despite random topographies having a cell shape that is most dissimilar to other topographies. This suggests factors in addition to cell shape contribute to cell fate.

Osteogenic media induced cell shape changes when cultured with flat substrates – with cell area significantly increasing for osteogenic conditions. This result suggests that cells cultured on 2D substrates will have relatively large areas when differentiating towards the osteogenic lineage. This finding is in line with previous literature demonstrating larger cell area for osteogenic cells – an effect regulated through increased intracellular forces^9^,^32^. However, this relationship is complicated by further observations. Large areas alone do not correlate to osteogenic function – as can be seen comparing aligned to flat topographies. The relatively larger cell area of aligned topographies does not correspond to the increased expression of osteogenic markers. Thus we conclude that osteogenesis is not correlated to shape parameters alone. This is likely a result of the complex relationship between stem cell fate and the extracellular environment. This echoes ambiguities discussed by Oh *et al*. where nanotopography regulated changes to osteogenic differentiation, but also resulted in changes to material properties including hydrophilicity and protein adsorption. It is additionally noted that changes to topography alter cell attachment density, with lower attachment for larger nanofeatures. Such changes obscure the fundamental regulator of such cell behaviors since the it is known that low seeding density increases osteogenic differentiation of MSCs^5^,^40^.

Stem cell differentiation can be driven by changes to the mechanical and architectural properties of the extracellular environment. In practice this is often achieved by changing substrate stiffness or surface properties^1^. While techniques to alter these properties are readily available and simple to implement, the relationship between a cells physical environment and its behavior is complex. There are many examples of materials that present different cell behaviors in response to similar physical environments. For example, materials with aligned topography show conflicting reports with respect to their ability to regulate osteogenesis, with results supporting both upregulation^11^,^12^,^41^ and no significant change^42^,^43^ furthering the debate. Here, we show osteogenesis of ASCs is not regulated by either flat, random or aligned nanotopography. Contrary to these findings, a study by Kim *et al*., demonstrates that aligned topographies upregulate osteogenesis in MSCs^28^. While, this study was limited to a comparison between aligned topographies and flat topographies, our study too included a flat topography control. In addition to this study, others have shown upregulation of osteogenesis. Namgung *et al*. demonstrated that MSCs cultured over aligned topographies upregulate osteogenesis relative to topographies with randomly oriented fibrous nanostructures. They extend their hypothesis to suggest that aligned topography regulates osteogenesis by increasing intracellular force. This claim is supported by upregulation in the expression of Ras homolog gene family, member A (*RHOA*), Rho-associated coiled-coil containing protein kinase 2 (*ROCK2*) and focal adhesion kinase (*FAK –* or protein tyrosine kinase 2 *PTK2*) – genes implicated in the transfer of intracellular force and regulation of cytoskeletal mechanotransduction. Contentiously, the authors state that the increased expression of these markers is due to the relatively larger area of cells cultures on aligned topographies and thus increased intracellular forces. Yet, cell area was not quantified in their study.

Myogenesis of ASCs is altered as a result of nanotopographical alignment. Specifically, gene expression analysis of ASCs cultured over random and aligned topographies shows myogenic markers *DES, PAX7, MYH2* and *MYF5* were upregulated relative to random topographies. However, the expression of *MYOD1*, a necessary marker for commitment towards the myogenic lineage was not upregulated^44^. Dang *et al*. reported similar gene profiles for the differentiation of MSCs when comparing cultures between aligned electrospun nanofibers and flat substrates. However, unlike our study, this report does not examine the effects of fiber orientation, but rather compares aligned substrates to flat ones^16^. Similar to our results, they showed that aligned topography did not alter osteogenic differentiation, but did upregulate myogenesis with upregulation of *DES* and *PAX7* (as well as paired box 3 (*PAX3*), collagen type 4 (*COL4*) and myogenic factor 4 or myogenin (*MYOG*) and no change to the expression of *MYOD1*. The authors concluded that their substrates upregulated myogenesis, although the induced commitment was possibly transient and required further myogenic signaling for complete differentiation. We speculate this is a likely scenario for ASC differentiation to muscle progenitor cell in the present study. Similar to the report by Dang *et al*. and the results herein, Jana *et al*. compared the effects of aligned microchannels and flat films in a chitosan-polycaprolactone hydrogel substrates and showed that aligned features induced cell alignment and upregulated the expression of *MYOG* but did not alter *MYOD1* expression^45^.

The influence of aligned topography on cell morphology and myogenesis is widespread^17^,^18^,^22^,^23^,^24^,^25^. Similar to this work, Cooper *et al*. compared fibers of random and aligned orientations^23^. While their study did not use stem cells, they showed increased attachment and proliferation of C2C12 myoblast cells over topographies with aligned fibers. Further, qRT-PCR showed aligned fibers upregulated *MYOG*, troponin and myosin heavy chain expression relative to substrates with randomly orientated fibers. A study by Choi *et al*. studied the effects of substrate type on myogenesis of ASCs. This study did not compare the effects of orientated topographies, but rather compared substrates without patterning to those with alternating channels of high and low stiffness polyacrylamide (similar to comparing flat and aligned topographies)^24^. Patterning of the underlying substrate was reflected in the organization and morphology of ASCs. Cells preferentially adhered to the stiff regions of the substrates aligning with the direction of the channels, increasing cell aspect ratio and enhancing myotube formation. However, no further functional analysis of stem cell differentiation was conducted. Yang *et al*. compared aligned to flat topographies along with the effects of coating these topographies with gold and titanium. While aligned topographies induced alignment of C2C12 myoblasts relative to flat topographies they did not alter the expression of *MYF5, MYOG* or *MYOD1*. However, coating the substrates with conductive films of gold and titanium did upregulate expression of these markers. The authors attribute this to the changes in inherent conductivity – a property known to upregulate myogenesis as reported elsewhere^38^,^46^,^47^. It is possible that the regulatory effects of conductivity on differentiation are seen herein. Both random and aligned topographies are considerably more conductive than flat coverslip control topographies, and both see upregulation of the myogenic marker *PAX7* at 14 and 21 days. However, in addition to *PAX7*, aligned topography also upregulates *MYF5* at 14 days and *MYF5* and *DES* at 21 days. Given that the contact angle and conductivity of random and aligned topographies are the same; P = 0.84 (See Supplementary Information), aligned topography (alignment of conductive CNTs) does upregulate myogenesis – suggesting that the physical and electrical properties of the extracellular environment work together to regulate cell differentiation.

Previous reports have shown topography can induce similar changes in the expression of mRNA to that observed herein. Such an expression profile is similar to that of muscle progenitor cells in a quiescent undifferentiated state in which myogenesis is arrested and cells are free to proliferate assisting muscle regeneration. Such satellite cells are a distinct myogenic cell lineage responsible for postnatal growth and repair of muscle. These cells are characterized by the suppression of *MYOD1* and an overexpression of *PAX7* – as seen in our results^48^. Further, such high expression of *PAX7* has been hypothesized to not only specify satellite cell lineage but also restrict alternative developmental programs^49^. Together these results suggest that ASCs cultured on aligned topographies differentiate to the muscle progenitor satellite cell state.

Future efforts to understand the relationship between substrate topography, its influence on cell shape and the mechanotransductive machinery of the cell should examine a wider range of phenotypes. Using the characterization techniques herein, fluorescent time-lapse imaging can be employed to examine dynamic cell shape behavior over a wider range of phenotypes. Together with machine learning techniques (that can be used to establish relationships between properties of differing populations, such as statistical classification methods) clear relationships and predictions may be made between phenotype and dynamic cell shape properties. A deeper understanding of these properties together with advances in nanofabrication techniques such as photolithography and 3D printing could be used to fabricate topographies that better drive cell differentiation in physiological settings.

## Methods

### Substrate fabrication

Detailed account of substrate fabrication can be found in the supplementary information. Figure S1 shows a flow diagram of the synthesis procedure. Briefly, substrates with aligned and random nanotopographies were fabricated by coating the surface of glass coverslips with carbon nanotubes (CNTs). Aligned topographies were fabricated by coating glass coverslips with CNTs from a drawable forest of aligned CNTs^29^. Sheets of aligned CNTs were dry drawn directly from the forest and layered the over the top of 18 mm circular glass coverslips (see Figure S1). Propanol was then dripped densifying the CNT sheet onto the cover slip such that surface tension forces would cause the CNTs to adhere to the cover slip. To create substrates with random nanotopography, CNTs were removed from the drawable forest and dispersed into a solution of propanol with 0.1 wt% polyvinylpyrrolidone and bath sonicated (20 W for 2 hours). Following sonication 160 μL of this solution was pipetted onto coverslip surfaces and dried in a vacuum oven. To remove PVP and carbonaceous impurities from the coverslips, substrates of both types of nanotopographies as well as uncoated coverslip controls were placed in an oven and oxidized at 400 °C for 2 hours and then pyrolysed in argon at 600 °C overnight. The areal density of random substrates was matched to that of aligned substrates measuring the absorbance through the different substrates (Figure S2). Absorbance through aligned and random substrate types, measured using a NanoDrop 1000 (Thermo Scientific), was found to be equivalent when a CNT solution of 31.3 μg mL^−1^ was used (an areal density of ~2.7 μg cm^−2^ – a result comparable to previously reported values for sheets pulled from drawable ACNT forests)^29^. Prior to experimentation all surfaces were immersed in 70% ethanol overnight. Likeness between random and aligned substrate types was confirmed with SEM, conductivity measurement (with 4-point probe) and contact angle. Figure S3 shows a histogram of CNT diameter as observed over aligned and random topography with SEM (FESEM) (Zeiss Ultra Plus, secondary electron detector).

### Cell Culture

Adipose-derived stem cells (Invitrogen) were isolated from liposuction samples of 3 donors and cultured as per manufacturers instructions in MesenPRO RS Basal Medium with the supplementations of 2 mM L-glutamine and MesenPRO RS Growth Supplement used until passage 4. Media was then changed to ‘ASC expansion media’; α-MEM (Gibco), supplemented with 10% (v/v) heat-inactivated fetal calf serum (FCS, Gibco), 1% penicillin/streptomycin (Gibco). ASCs at passage five were used for the study. For all studies ASCs were cultured at a density of 10,000 cells cm^−2^ in standard conditions 37 °C, 95% humidity and 5% CO_2_. The pro-osteogenic media used has been widely reported^50^. Expansion media supplemented with ascorbic acid-2 phosphate (50 μg mL^−1^), β-glycerophosphate (10 mM) and dexamethasone (10 nM)). Figure S5 shows qRT-PCR and Alizarin Red staining comparing expansion and osteogenic media types. During experimentation media was changed every 3 days.

### Alizarin Red Staining

Alizarin Red S Staining was carried out as previously described^51^. In brief, at 21 days cells were incubated with 70% ethanol for 5 minutes. They were then washed with DI water and incubated in Alizarin Red S Solution (pH 4.3) for 30 minutes at room temperature. Then cells were washed in water four times. Microscopy was completed on an inverted bright field light microscope (CKX41, Olympus).

### PCR

qRT-PCR was used to evaluate expression of mRNA over the different substrates. Total RNA was isolated using Trizol (Sigma-Aldrich, USA) and purified with a RNeasy Mini Kit (Qiagen) according to the manufacturer’s instructions. First-strand cDNA was synthesized from 1 μg of RNA using a Tero cDNA synthesis kit (Bioline) according to the manufacturer’s instructions. Osteogenic genes, including *18 S, OPN, RUNX2, COL1A1, ALP,* and *BMP2* were amplified in 20 μL volume per reaction including 10 μl of SYBR green real-time PCR master mix (Applied Biosystems), 0.5 μl for each forward and reverse primer (final concentration of 1 μM), 4 μl of DNase/RNase-free distilled water (Gibco) and 100 ng of cDNA in 5 μl and analyzed using a Rotor-gene 6000 (Corbett Life Science). To evaluate the expression of the myogenic genes *β2 M, RPL13A, DES, PAX7, MYH2, MYF5* and *MYOD1*, a total of 20 ng of cDNA for each experimental condition (flat, random and aligned topographies) was performed on Mx3000 P qPCR detention system (Stratagene). cDNA was amplified in a 15 μL volume per reaction including 7.5 μl of SYBR green real-time PCR master mix (Applied Biosystems), 0.15 μl for each forward and reverse primer (final concentration of 1 μM), 2.2 μl of DNase/RNase-free distilled water (Gibco) and 5 μl of cDNA sample. All reactions were performed in triplicate. Melting curves were generated for each real-time RT-PCR to verify the specificity of each PCR reaction. Relative gene expression was the calculated using the ∆∆CT method normalized with the house-keeping genes *18 S* for osteogenic markers and *β2 M* and *RPL13A* for myogenic markers. For osteogenic expression samples were calibrated to flat topography coverslip control group, where for myogenic markers samples were calibrated against reference human cDNA derived from mRNA extracted from different human tissues – providing broad gene coverage (Clontech). Primer sequences are shown in SI Table S1.

### Cell Transduction

Cells were transduced with Lifeact-GFP (kind gift from R. Wedlich-Söldner, University of Münster, Germany). Subsequent to transduction, cells were sorted for double GFP and mCherry fluorescence on a BD Influx flow cytometer and then expanded following standard protocols prior to use in experiments.

### Flow Cytometery

Transduced and non-transduced ASCs were collected by trypsin/EDTA digestion, washed twice with fluorescence-activated cell sorting buffer (2% fetal calf serum, 2 mM EDTA and 0.02% sodium azide in PBS) and incubated with 0.5 μg ml^−1^ 4,6-diamidino-2-phenylindole (Molecular Probes, Thermo Fisher Scientific) for exclusion of dead cells. A total of 20,000 events for each population of ASCs were collected on a BD Biosciences Fortessa flow cytometer. GFP-Lifeact was excited using the 488 nm laser and collected in the B430/30 channel; Data was analyzed with FlowJo software.

### Time-lapse Imaging

Time-lapse imaging was completed using the live-cell imaging equipment IncuCyte ZOOM (Essen Bioscience). Nunc 24-well plates were milled with an 18 mm end mill to remove the base of the culture wells (Figure S2). Coverslips were placed in the recess left by the mill and glued in place using Sylgard 184 (Dow Corning) and left to cure for 3 days. Following this period, the well plates were washed with sterile deionized water to ensure any uncured solutions were removed. ASCs stably expressing LifeAct-GFP and CAAX-mCherry were used at passage 5. To assist with image segmentation, these cells were diluted with wild type ASCs to lower the density of cells expressing fluorescent markers. This resulted reduced the changes of cells overlapping and thus poor image segmentation. Images were taken over the course of 21 days. To capture different cell behaviors while maintaining manageable amounts of data the frequency at which images were taken was altered throughout the 21 days period of imaging. During the first 6 hours of the experiment (0 to 6 hours) images were captured every 10 min. From 6 to 96 hours (4 days) images were captured every 15 min. From 96 to 504 hours (or 4 to 21 days), images were captured every 1 hour. Images were taken across 4 different conditions. This included flat coverslip topographies with expansion media, random nanotopography, aligned nanotopography and uncoated flat coverslip with osteogenic media (flat-osteo). The experiment was run in duplicate with 4 locations imaged over each substrate.

### Image segmentation and processing of cell shape metrics

To characterize cell shape in an unbiased fashion, time-lapse microscopy data was processed using CellProfiler analysis software. Image processing pipeline can be found in SI Table S3. Demonstration of a typical image segmentation can be found in the SI Figure S7. For all segmented objects in an image, the CellProfiler Module ‘MeasureObjectSizeShape’ was used to extract metrics on cell area and shape; more information on these metrics can be found at the CellProfiler website: http://www.cellprofiler.org/CPmanual/MeasureObjectSizeShape.html. Extracted features were then imported into MATLAB R2015b where data for all objects from the same substrate/time but different fields of view was combined and graphed. To validate that the extraction of shape metrics using CellProfiler could be automated, we compared objects that were manually segmented to objects that were obtained from automated segmentation with CellProfiler. We tested that automated segmentation of objects was equivalent to that done manually. This analysis was completed for the various cell shape metrics we examine throughout this work. Table S4 lists the models, correlation and P-values obtained when comparing metrics between manually and automatically segmented images.

### FFT Analysis

To quantify the alignment of cells we compared 2D FFT of images obtained from time-lapse microscopy. The power spectrum of the 2D FFT of an images can be used to compare the presence of features at a given orientation^52^. Summing the power spectrum of 2D FFT images at a given angle the prominence of cell features at given orientations is compared between the different substrate types. For each topography type, the power spectrums from each field of view at a given time point were combined and normalized. Following calculation of the power spectrums across the complete 21 days, spectrums for a given substrate were combined and graphed as a surface (as per Fig. 4B). Note that the measurement neglects angles between 180° to 360° since alignment at 90° and 270° are equal in FFT space – i.e. cells aligned north-to-south and south-to-north are indistinguishable. Additional details provided in supplementary information.

## Additional Information

**How to cite this article**: Newman, P. *et al*. Relationship between nanotopographical alignment and stem cell fate with live imaging and shape analysis. *Sci. Rep.* **6**, 37909; doi: 10.1038/srep37909 (2016).

**Publisher's note:** Springer Nature remains neutral with regard to jurisdictional claims in published maps and institutional affiliations.

## Supplementary Material

Supplementary Information

## Figures and Tables

**Table 1:** Hours taken from cell seeding for cell shape metric to vary between two topographies.

	Flat/Flat-osteo	Flat/Aligned	Random/Flat	Aligned/Random
Area	0.5	1.5	0.5	0.5
Circularity	N/A	N/A	25.5	5.25
Major Axis	1.25	1.25	1.5	1.5
Minor Axis	0.5	0.5	0.5	0.5

Shows the number of hours taken for cell shape to be dissimilar when attaching on different topographies and spreading from an initial unattached round state. Different columns compare flat/flat-osteo, flat/aligned, random/flat, and aligned/random for the different cell shape parameters area, circularity, major axis and minor axis. N/A indicates the parameters never diverge. Additional information on the analysis is available in the supplementary information.
