# Corrigendum: Relationship between nanotopographical alignment and stem cell fate with live imaging and shape analysis

**DOI:** 10.1038/srep43073

**Published:** 2017-03-07

**Authors:** Peter Newman, Jorge Luis Galeano Niño, Pamela Graney, Joselito M. Razal, Andrew I. Minett, João Ribas, Raquel Ovalle-Robles, Maté Biro, Hala Zreiqat

Scientific Reports 6: Article number: 37909; 10.1038/srep37909 published online: 12 02 2016; updated: 03 07 2017.

The original version of this Article contained an error in the spelling of the author Jorge Luis Galeano Niño, which was incorrectly given as Jorge Luis Galenano-Niño.

Additionally, there were typographical errors in Affiliation 9 which was incorrectly listed as ‘Sydney Medical School, The University of Sydney, NSW, 2006, Australia.’ The correct affiliation is listed below:

Centenary Institute, Sydney Medical School, The University of Sydney, NSW, 2006, Australia.

These errors have now been corrected in the PDF and HTML versions of the Article.

