# Supplementary material for: Relationship between nanotopographical alignment and stem cell fate with live imaging and shape analysis
Source: Sci Rep. 2016 Dec 2;6:37909. doi: 10.1038/srep37909 (PMC5133629; doi:10.1038/srep37909)
Supplement: Supplementary Information [file srep37909-s1.pdf]

# Supporting Information

# Relationship between nanotopographical alignment and stem cell fate with live imaging and shape analysis

Peter Newman, Jorge Luis Galeano Niño, Pamela Graney, Joselito M. Razal, Andrew Minett, João Ribas, Raquel Ovalle-Robles, Maté Biro,* Hala Zreiqat*

**Fabrication of Substrates**

Substrates with aligned and random nanotopographies were fabricated by coating the surface of glass coverslips with carbon nanotubes (CNTs). Aligned topographies were fabricated by coating glass coverslips with CNTs from a drawable forest of aligned CNTs.[1] An aligned drawable CNT forest was provided by Joselito Razal and Raquel Ovalle-Robles. Self-supporting CNT sheets were dry drawn directly from the aligned forest. These Self-supporting MWCNT sheets are form as a highly anisotropic aerogel continuously and can be than can be densified into strong sheets that are as thin as 50 nm. These continuous CNT sheets have an areal density of about 2.7 $\mu g/cm^2$ and have no fundamental limit on sheet width or length (Figure S1). These CNT sheets were then directly deposited onto a 18 mm circular glass coverslip. Propanol was then dripped over the CNT sheet, densifying and adhering them on the coverslips due to capillary forces. To create substrates with random nanotopography, CNTs were removed from the drawable forest and dispersed into a solution of propanol with 0.1 wt % polyvinylpyrrolidone and bath sonicated. Following sonication 160 µL of this solution was pipetted onto coverslip surfaces and dried in a vacuum oven. To remove PVP and carbonaceous impurities from the coverslips, substrates of both types of nanotopographies as well as uncoated coverslip controls were placed in an oven and oxidized at 400 ˚C for 2 hours and then pyrolysed in argon at 600 ˚C overnight.

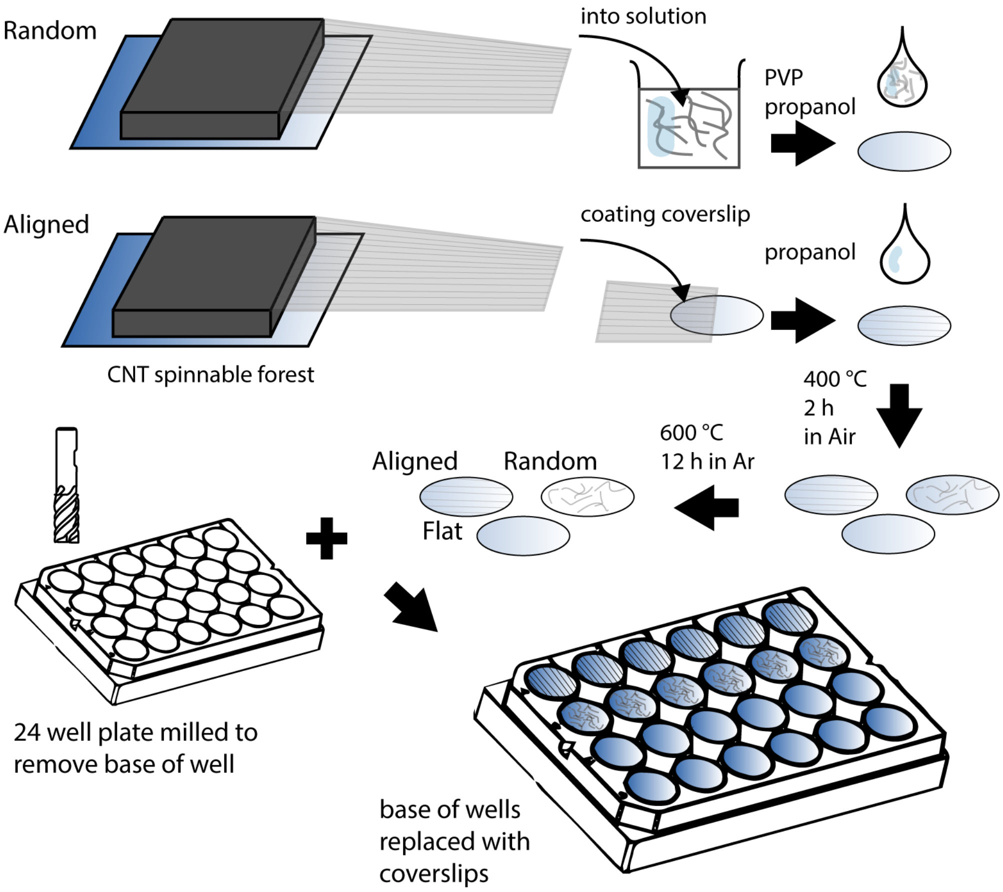


Figure S1 shows the fabrication scheme of CNT coated coverslips with aligned drawable forest of CNTs. The procedure for both random, aligned and flat topographies is shown.

The areal density of random substrates was matched to that of aligned substrates (Figure S2). Absorbance through aligned and random substrate types, measured using a NanoDrop 1000 (Thermo Scientific), was found to be equivalent when a CNT solution of 31.3 μg $mL^{-1}$ was used. This equates to an areal density of 2.7 μg $cm^{-2}$ – a result comparable to previously reported values for sheets pulled from drawable aligned forests[2]. All subsequent random substrates were prepared as described above with a coating of CNT solution at 31.3 μg $mL^{-1}$. All coverslip control surfaces including uncoated and fibronectin coated glass were treated with vacuum oven, oxidation and pyrolysis treatments described above. Prior to experimentation all surfaces were immersed in 70% ethanol overnight.

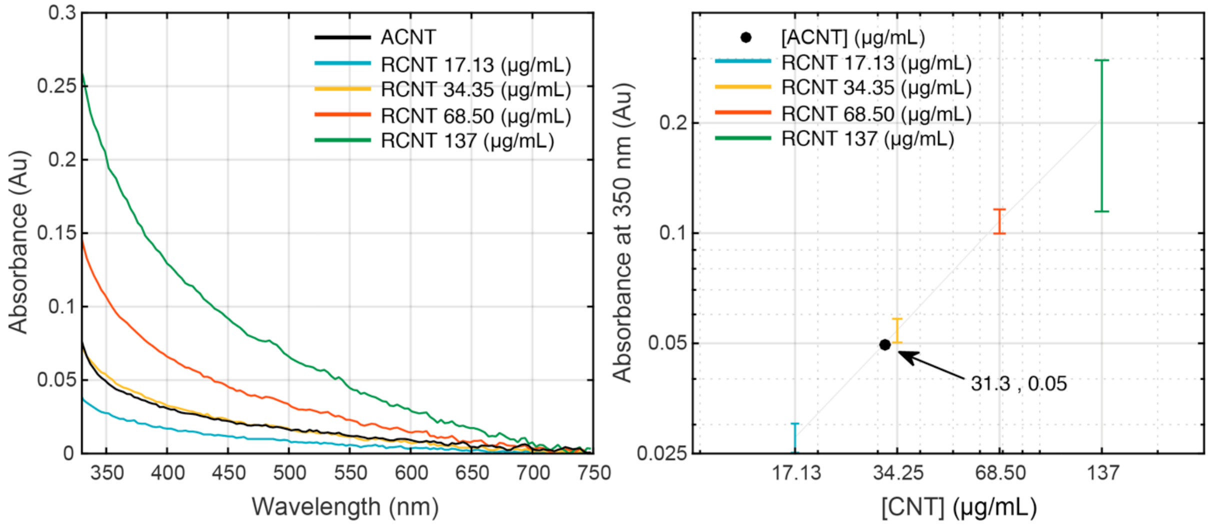


Figure S2: Absorbance of visible light through random coated substrates at varying concentration as compared to aligned coated substrates (left). Averaging results and plotting the absorbance at 350 nm against concentration of random topographies (or random carbon nanotubes (RCNT)) shows comparable areal density to aligned topographies (or aligned carbon nanotubes (ACNT)) coating at 31.3 μg $mL^{-1}$ (right)

The diameter of the CNTs on either substrate types was characterized to ensure mixing, dispersion, sonication, and coating processing had not altered the dimensionality of CNT populations between substrate types. Figure S3 shows histograms of the diameter of CNTs over aligned and random substrates where the mean diameter of 26.38 nm and 25.13 nm was comparable over aligned and random substrate types, respectively.

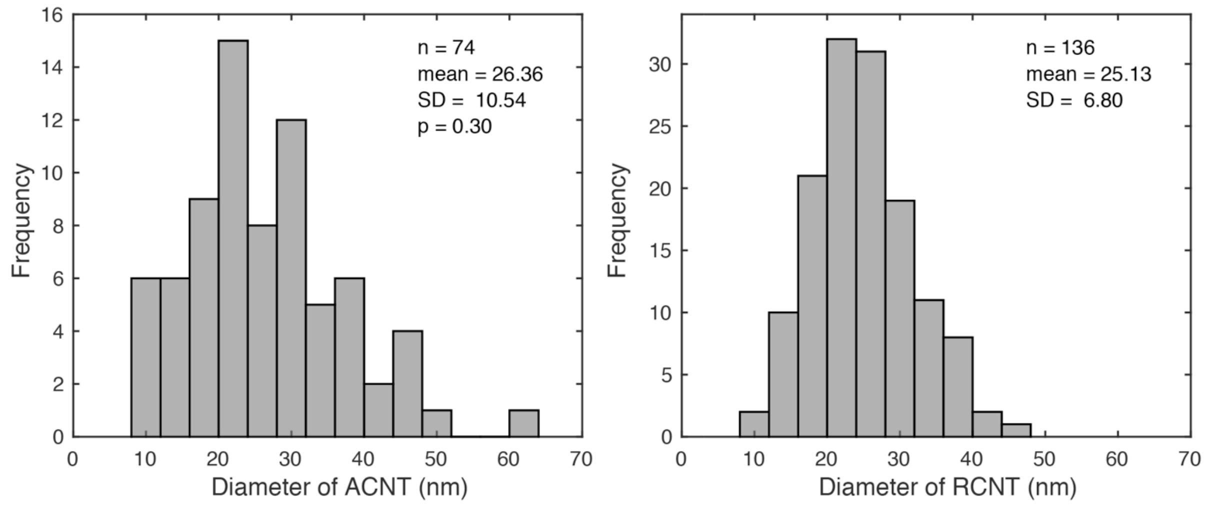


Figure S3: Histograms of diameters of (left) aligned carbon nanotubes (ACNT) and (right) random carbon nanotubes (RCNTs). Comparing groups: $P < 0.30$; unpaired two sample Student's t-test comparing diameters measured with SEM.

Measurements of water contact angle is shown in Figure S4A and B with 2 μL water droplet over random and aligned topographies, respectively (measured using a contact angle goniometer, VCA2000 (AST Inc.)). Substrates were comparable (Figure S4C) with mean contact angle of 89.143 (SD = 2.113, n = 8) for aligned topographies and 90.015 (SD = 4.017, n = 8) for random topographies; $P = 0.58$ (unpaired two sample Student's t-test). The sheet resistance of the substrates was measured using the 4-point

probe / Keithley SCS-4200 (Figure S4D). Mean sheet resistance of 2997 Ω $sq^{-1}$ was measured for aligned topographies as compared to 3111 Ω $sq^{-1}$ for random topographies; P = 0.84 (unpaired two sample Student's t-test)

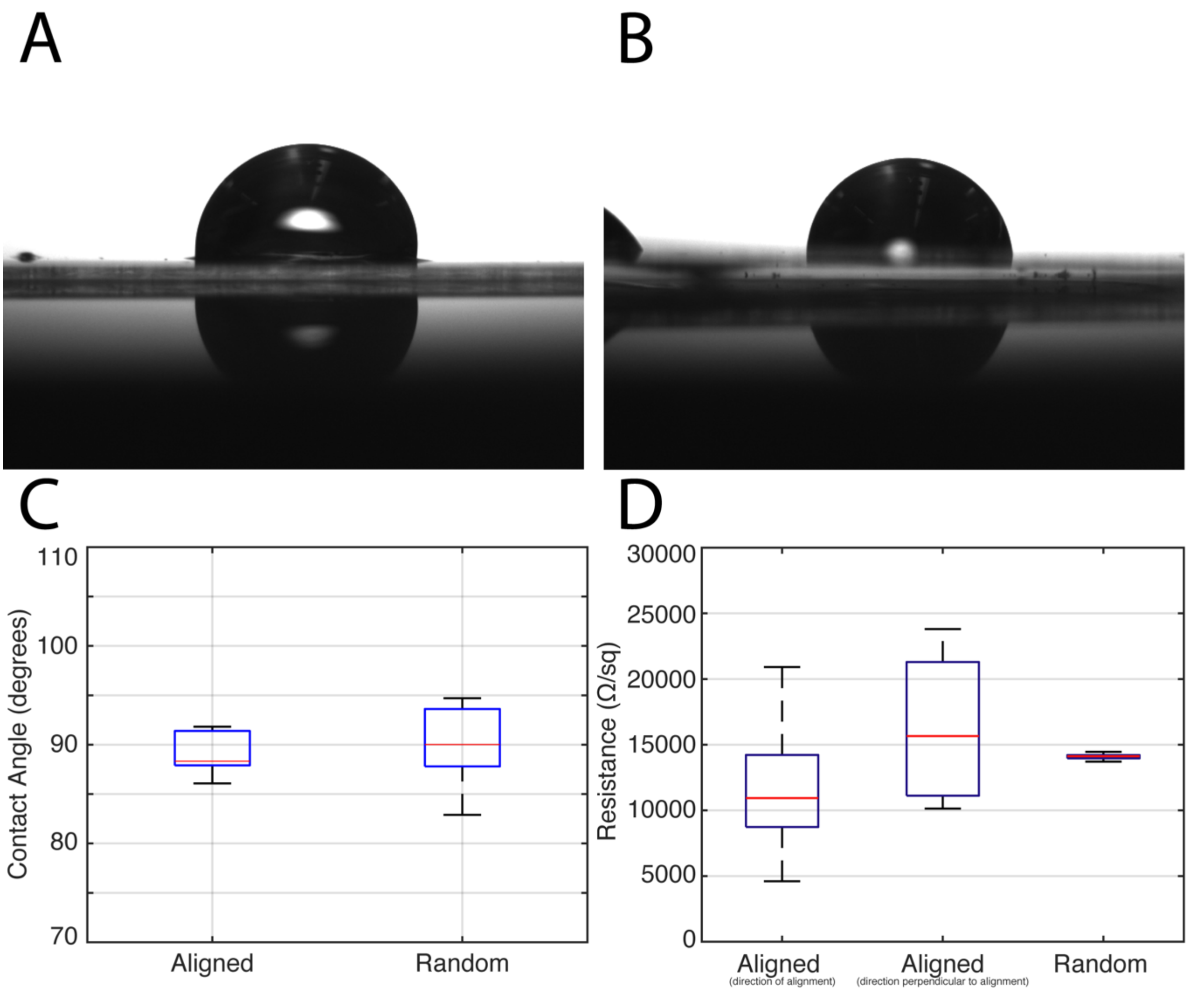


Figure S4: Measurements of water contact angle is shown in panels A and B with 2 μL water droplet over random and aligned topographies. Water contact angle data is summarized in panel C comparing random and aligned topographies. Panel D compares the sheet resistance of the substrates measured using a 4-point probe.

Osteogenic behavior was induced in ASCs cultured in pro-osteogenic media defined as expansion media supplemented with ascorbic acid-2 phosphate (50 μg $mL^{-1}$), β-glycerophosphate (10 mM) and dexamethasone (10 nM)). Figure S5 shows RT-qPCR and Alizarin Red staining in ASCs cultured in expansion or osteogenic media. Panels B and C compare red stained calcific deposits of osteogenic and expansion media respectively with prominence of the red staining is far higher for osteogenic media. Panel A shows RT-qPCR results comparing expression of the osteogenic markers *ALP*, *COL1A1*, *OPN*, *RUNX2* and *BMP2*. At 21 days the expression of *COL1A1* and *OPN* is significantly higher for osteogenic media. Together these results confirm the positive differentiation function of the osteogenic media when used to drive osteogenesis of ASCs.

Table S1: Primers used for Real-Time Polymerase Chain Reaction

| Primer | Sequence (5'- 3') | Melting Temperature (°C) |
|---|---|---|
| ***18S*** | F GTAACCCGTTGAACCCCATT | 60 |
| | R CCATCCAATCGGTAGTAGCG | |
| ***OPN*** | F TTCCAAGTAAGTCCAACGAAAG<br>R GTGACCAGTTCATCAGATTCAT | 60 |
| ***RUNX2*** | F ATGCTTCATTCGCCTCAC<br>R ACTGCTTGCAGCCTTAAAT | 60 |
| ***COL1A1*** | F ATCTCCTGGTGCTGATGGAC<br>R GCCTCTTTCTCCTCTCTGACC | 60 |
| ***ALP*** | F CGTGGCTAAGAATGTCATCATGTT<br>R AGGGGAACTTGTCCATCTCC | 60 |
| ***BMP2*** | F AGTTGCGGCTGCTCAGCATGTT<br>R CCGGGTTGTTTTCCCACT | 60 |
| ***PAX7*** | F ACAAGAGGGAAAACCCAGGC<br>R CGCGGCTAATCGAACTCACT | 60 |
| ***MYF5*** | F CGCCTGAAGAAGGTCAACCA<br>R TGTAGCGGATGGCATTCCTG | 60 |
| ***MYOD1*** | F ATGACGACCCGTGTTTCGAC<br>R AGTGCTCTTCGGGTTTCAGG | 60 |
| ***MYH2*** | F TACTGCACACCCAGAACACC<br>R TTTTCTTCTGCATTGCGGGC | 60 |
| ***DES*** | F GAGACCATCGCGGCTAAGAA<br>R GGGCGTCGTTGTTCTTGTTG | 60 |
| *18S, 18S ribosomal RNA; OPN, osteopontin; Runx2, runt-related transcription factor 2; Col1A, Collagen 1A; ALP, alkaline phosphatase; BMP2, bone morphogenetic protein 2; PAX7, paired box protein 7; MYF5, myogenic factor 5; MYOD1, myogenic differentiation 1; MYH2, myosin heavy chain 2; DES, Desmin protein* | | |

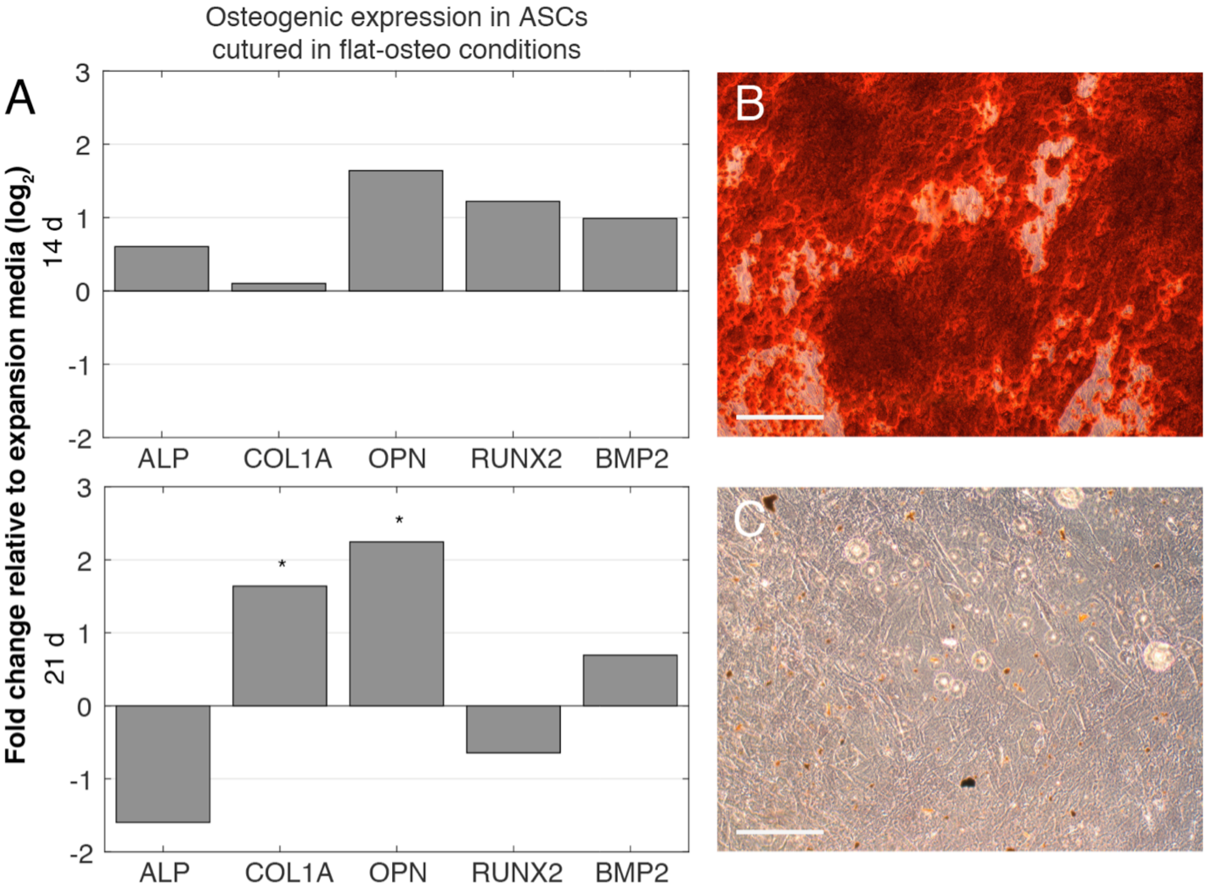


Figure S5: A) RT-qPCR results for osteogenic gene expression of pro-osteogenic media. ASCs were cultured in expansion media: αMEM, 10% FCS and 1% pen/strep or an osteogenic media with pro-osteogenic factors: expansion media supplemented with ascorbic acid-2 phosphate (50 µg $mL^{-1}$), ß-glycerophosphate (10 mM) and dexamethasone (10 nM)). * shows significance compared to expansion media; P < 0.05 (unpaired two sample Student's t-test). Alizarin red staining of ASCs cultured in B) osteogenic and C) expansion media (scale bar 100 µm)

**Transduction with LifeAct**

Cell shape parameters were obtained using fluorescent imaging of ASCs stably expressing LifeAct-GFP (an 17-amino-acide peptide that stains F-actin without interfering with actin dynamics - kind gift from R. Wedlich-Söldner, University of Münster, Germany).[3] Transduction was confirmed with fluorescent imaging and flow cytometry (Figure S6). Subsequent to transduction, cells were sorted for double GFP on a BD Influx flow cytometer and then expanded following standard protocols prior to use in experiments. GFP-Lifeact was excited using the 488 nm laser and collected in the B430/30 channel; Data was analyzed with FlowJo software.

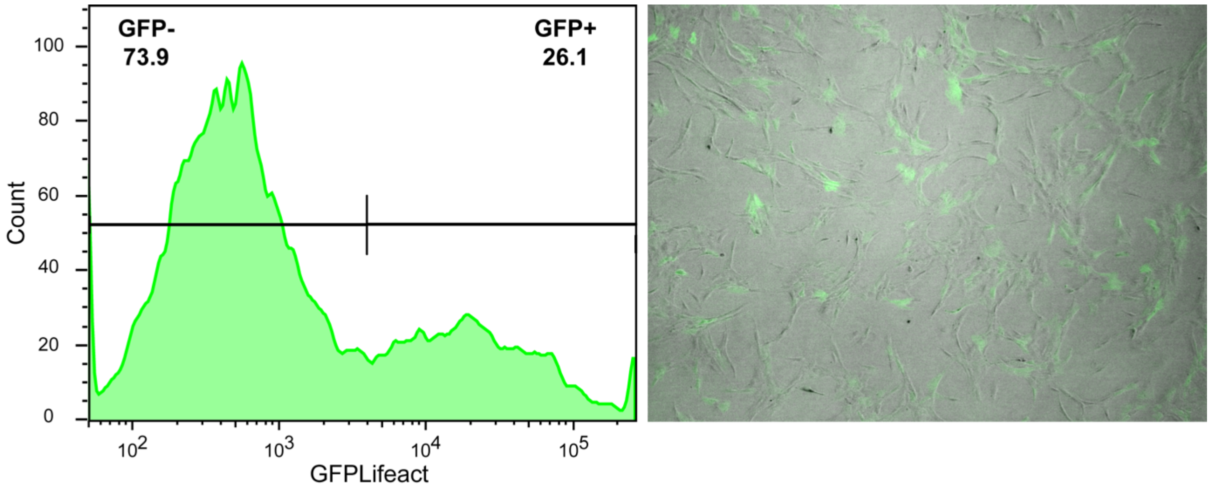


Figure S6: Left) Histogram of flow cytometry data showing ~26% ASCs expressing LifeACt-GFP. Right shows fluorescent imaging of ASCs following transduction with LifeAct-GFP.

**Imaging**

Time-lapse imaging was completed using ASCs expressing LifeAct-GFP with the live-cell imaging equipment IncuCyte ZOOM (Essen Bioscience). Nunc 24-well plates were milled with an 18 mm end mill to remove the base of the culture wells (Figure S1). Coverslips were placed in the recess left by the mill and glued in place using Sylgard 184 (Dow Corning) and left to cure for 3 days. Following this period, the well plates were washed and soaked with sterile deionized water overnight to ensure any uncured solution was removed. ASCs stably expressing LifeAct-GFP and CAAX-mCherry were used at passage 5 at a density of 10,000 cells $cm^{-2}$. These cells were diluted with wild type ASCs to minimize the the chances of overlapping cells and thus poor image segmentation and extraction of cell shape parameters. Images were taken over the course of 21 days. To better capture the relatively fast process of adhesion the frequency at which images were taken was altered throughout the 21 day period of imaging. During the first 6 hours of the experiment (0 to 6 hour) images were captured every 10 minutes. From 6 to 96 hours (4 days) images were captured every 15 minutes. From 96 to 504 hours (or 4 to 21 days), images were captured every 1 hour. Images were taken across 5 different conditions. This included the 4 different substrate types with flat topography untreated coverslip and complete expansion media, random nanotopography, aligned nanotopography and flat untreated coverslip with osteogenic media (flat-osteo). The experiment was run in duplicate with 4 locations imaged over each substrate. Table S2 below lists and defines the cell shape parameters used in this study.

Table S2: Definitions of cell shape parameters

| | |
|---|---|
| Cell Area | We express cell area as the two-dimensional space filled by a given cell in $\mu m^2$ |
| Circularity | Circularity of a cell as the scaled ratio of it's area and perimeter – equal to $4\pi\text{Area}/perimeter^2$; equal to 1 for a perfectly circular object and decreases towards 0 for shapes with increasing perimeter for a given area. |
| Major Axis | The major axis of the cell is the longest axis of the smallest ellipse that completely encloses a cell. |

| | |
|---|---|
| Minor Axis | The minor axis of the cell is the shortest axis of the smallest ellipse that completely encloses a cell. |

**CellProfiler image segmentation process**

To characterize cell shape in an unbiased fashion, time-lapse microscopy data was processed using CellProfiler analysis software. Image processing pipeline is shown below in Table S3.

Table S3: Image processing pipeline

| **Process** | **Notes** |
|---|---|
| Original Image | Green fluorescent channel (LifeAct-GFP) taken from Incucyte ZOOM, encoded 16-bit, '.tif', RGB to maximize quality |
| ColorToGray | Creates grayscale image – converted for downstream thresholding and segmentation |
| RescaleIntensity | Image intensity rescaled (divide by image maximum) for consistent thresholding and segmentation |
| EnhanceOrSupressFeatures | Suppression of features sizes lower than 3 pixels. Removes speckles. |
| Smooth | Smooth features with Gaussian filter 3 pixels |
| CorrectIllumination Calculate | Calculates and corrects image for illumination artifacts, calculated for each image from the background using splines method with automatically calculated spline parameters |
| RescaleIntensity | Image intensity rescaled (divide by image maximum) for consistent thresholding and segmentation |
| ImageMath | Illumination function calculated above is subtracted from the image, values less than 0 are set to 0. |
| IdentifyPrimaryObjects | Segmentation process was completed using maximum correlation thresholding (MCT) with an adaptive window.<br><br>Specifically the following parameters were set:<br><br>• Cells outside of the diameter range: 20 < diameter < 4000 are rejected<br>• Cells touching the border are rejected<br>• Adaptive MCT thresholding was used with automatic smoothing and a threshold correction value of 0.7<br>• An adaptive window size was set to 400 pixels<br>• Objects were separated set by ther intensity<br>• With a declumping smoothing filter size of 10 pixels<br>• Declumping was suppressed if local maxima were less than 15 pixels<br>• Image was not downsized from declumping |
| MeasureObjectSizeShape | Object shape parameters we measured |
| SaveImages | Grayscale images of objects (each object a different intensity) were saved for processing. |
| ExportToSpreadsheet | Object shape parameters we extracted into a spreadsheet. |

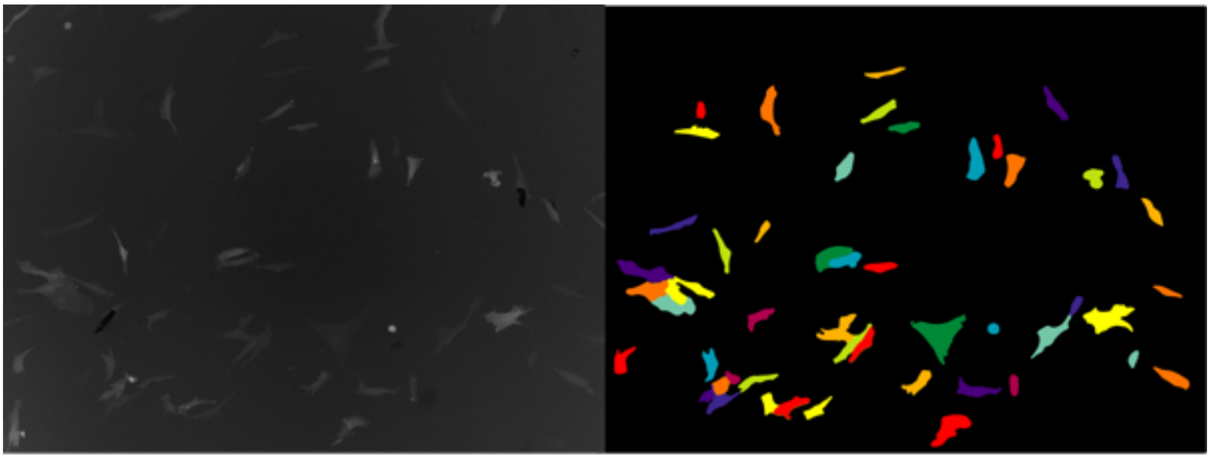

Figure S7: left shows fluorescent imaging of ASCs following transduction with LifeAct-GFP. Image at right shows segmention as applied by CellProfiler software.

An example of an image before and post segmentation is shown in Figure S7. For all segmented objects in an image, the CellProfiler Module 'MeasureObjectSizeShape' was used to extract metrics on cell area and shape; more information on these metrics can be found a the CellProfiler website: http://www.cellprofiler.org/CPmanual/MeasureObjectSizeShape.html. Extracted shape metrics were then imported into MATLAB R2015b where data for all objects from the same substrate and time but different fields of view were combined (time series plots in main article Figure 5 and 6).

Using a hampel filter, the quality and focus of each image was assessed prior to further analysis. The hampel filters was set to compute the median and standard deviation of a window composed of 10 adjacent data point (5 on either side). Data from images was rejected if the number of cells differed from the median of more than 5 standard deviations. Consequently, gaps in the data can be observed were the microscope was unable to focus correctly (see Figure S8).

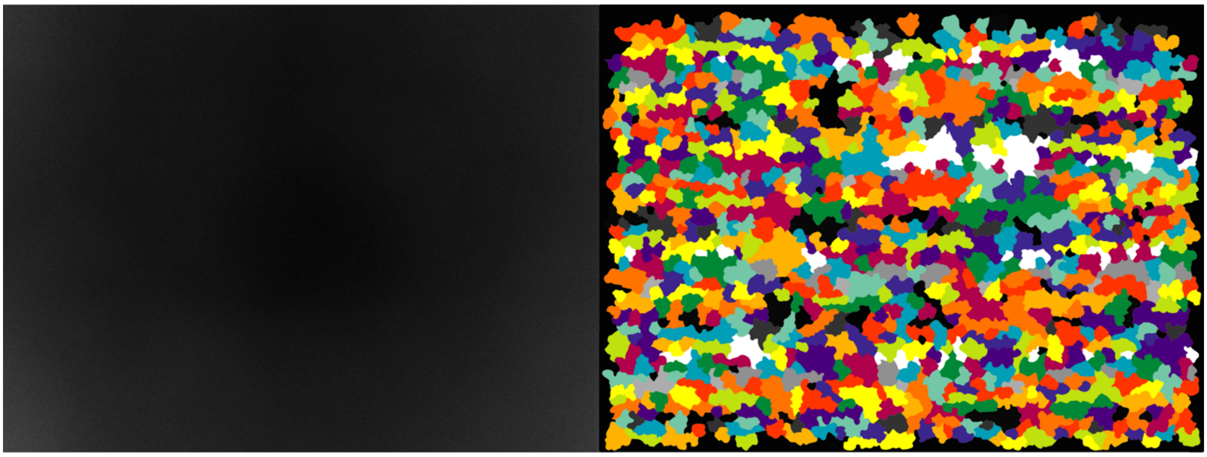

Figure S8: example of out of focus image and respective segmentation as applied by CellProfiler

To validate that the extraction of shape metrics using CellProfiler could be automated, we compared objects that were manually segmented to objects that were obtained from automated segmentation with CellProfiler. We tested that automated segmentation of objects was equivalent to that done manually.

To validate that image analysis could be automated we compared data which had been manually selected to that obtained from automated image analysis. Fitting the dynamic cell area to a power law model $area = \alpha_1 \cdot time^{\beta_1}$, as previously developed,[4] we determined the probability that the data sets

were equal using a F-test. This suggested that the null hypothesis was false (p ~ 0); and automated selection of cell masks can not be used to replace selection done manually (F score can be found in Table S4). However, inspection of the two data sets suggests a high degree of correlation between $\partial Area/\partial time$. Further, data from automated analysis yielded consistently lower values relative to data manually selected. To correct for this difference, we calculated a constant *the correction factor* (*CF*) using least square method: *0* = $(\sum_{time=0}^{time=time_{max}}(Area_{Manual} - CF \times Area_{Automated})^2$. Scaling data obtained through automated selection by the correction factor constant observed that the two sets of data closely fit each other (Figure S9A-Dii). Further reevaluation of the data with a F-test we demonstrated that the null hypothesis was true (p ~ 1); and both automated and manually selected data sets can be modelled with one equation. Using the same methods we completed analysis comparing the other metrics used herein. Table S4 lists the models, coefficients of trend line fits, F-scores and p-values obtained when comparing metrics between manually and automatically segmented images. Note that the model for circularity $(= \alpha_2 \cdot time^{(\beta_1 - 2 \cdot \beta_1 \cdot \beta_2)})$ was developed through substitution and simplification of the area $(= \alpha_1 \cdot time^{\beta_1})$ and perimeter $(= \alpha_{perimeter} \cdot time^{\beta_2})$ – developed elsewhere[4] – into the expression for circularity $4\pi \cdot area/perimeter^2$.

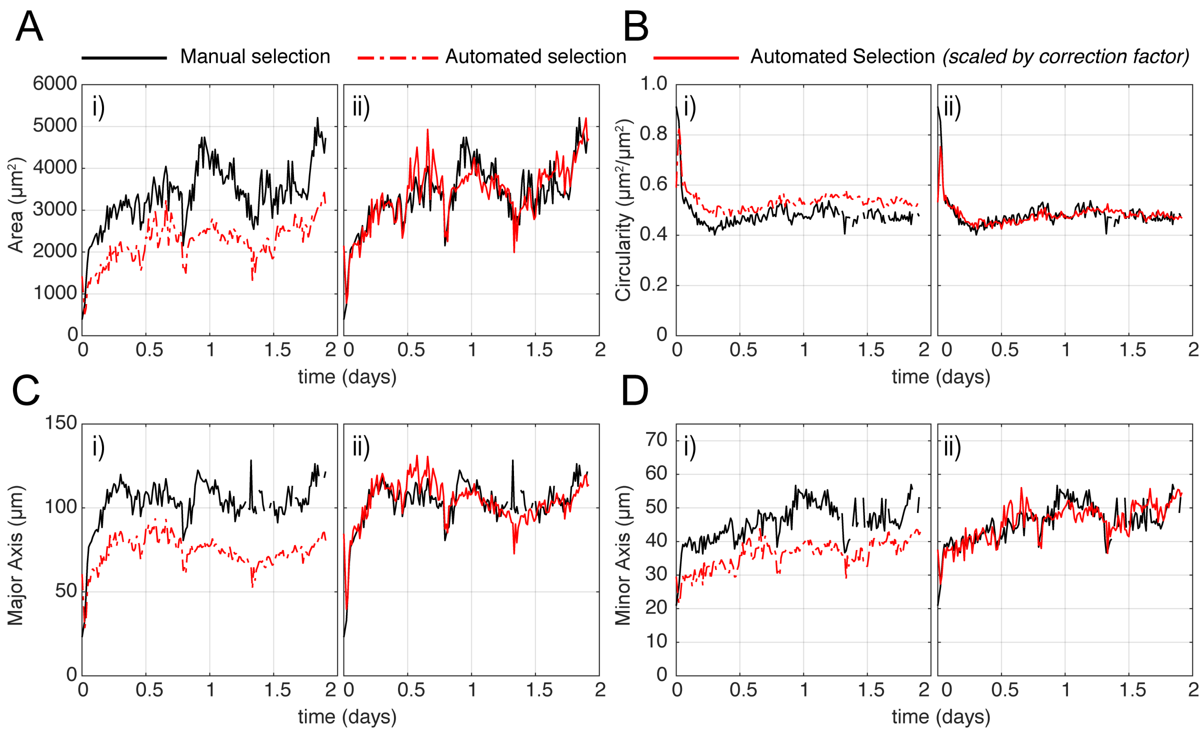


Figure S9: Comparison of time series plots showing various cell shape parameters of cells for manual selected, automated selection and automated selection scaled by the correction factor.

Table S4: F-test coefficients and scores for time series plots in Figure S9

| Data | Model | Co-efficient | | | F-score | p-value |
|---|---|---|---|---|---|---|
| **Area** | | | | | | |
| Manual | $\alpha_1 \cdot time^{\beta_1}$ | a = 3543 | b = 0.164 | | | |
| Automated | | a = 2356 | b = 0.174 | | | |
| Manual + Automated | | a = 2950 | b = 0.168 | | 317.7 | 0 |
| CF*Automated | | a = 3489 | b = 0.174 | | | |
| Manual + CF*Automated | | a = 3516 | b = 0.169 | | 0.663 | 0.999 |
| **Circularity** | | | | | | |
| Manual | $\alpha_2 \cdot time^{(\beta_1 - 2\beta_1\beta_2)}$ | a = 0.528 | b1 = 0.030 | b2 = 0.9783 | | |
| Automated | | a = 0.473 | b1 = 0.261 | b2 = 0.5675 | | |
| Manual + Automated | | a = 0.501 | b1 = 0.146 | b2 = 0.6093 | 55.08 | 0 |
| CF*Automated | | a = 0.478 | b1 = 0.139 | b2 = 0.6042 | | |
| Manual + CF*Automated | | a = 0.476 | b1 = 1.009 | b2 = 0.5159 | 0.361 | 1 |
| **Major Axis** | | | | | | |
| Manual | $\alpha_3 \cdot time^{\beta_3}$ | a = 105.9 | b = 0.061 | | | |
| Automated | | a = 75.06 | b = 0.032 | | | |
| Manual + Automated | | a = 90.48 | b = 0.049 | | 448.0 | 0 |
| CF*Automated | | a = 104.2 | b = 0.032 | | | |
| Manual + CF*Automated | | a = 105.1 | b = 0.047 | | 0.286 | 1 |
| **Minor Axis** | | | | | | |
| Manual | $\alpha_4 \cdot time^{\beta_4}$ | a = 47.19 | b = 0.095 | | | |
| Automated | | a = 37.16 | b = 0.106 | | | |
| Manual + Automated | | a = 42.18 | b = 0.100 | | 381.8 | 0 |
| CF*Automated | | a = 47.41 | b = 0.106 | | | |
| Manual + CF*Automated | | a = 47.30 | b = 0.101 | | 0.535 | 1 |

To compare the shape metrics of different topographies throughout the culture period we used a F-test over segments of time shorter than the duration of the study. For these comparisons we assumed that the data could be segmented and the data could be fit to a linear model. Figure S8 below shows the comparisons for time segments over which different topographies were statistically the same.

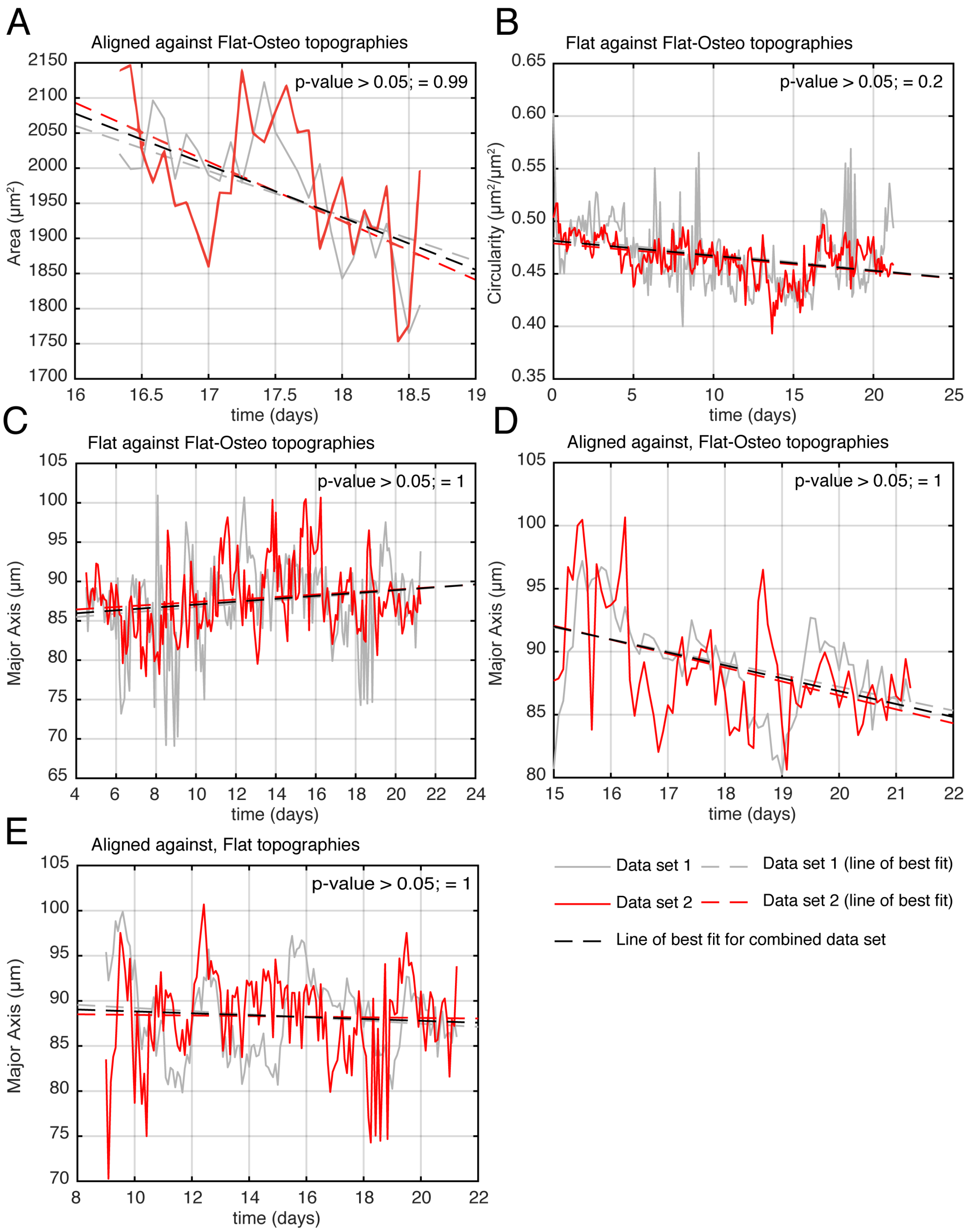


Figure S10: F-tests comparing time series plots of different topographies for short segments of data. Assumes short segments can be fit with a linear model.

**Testing for similarity between topographies for a given cell shape parameter**

Given that all cells begun with a similar non adherent circular shape, we examined the time taken for cell shape to become dissimilar between different topographies. Table 1 in the main manuscript summarizes this data. To obtain these values we truncated our data to cover short subsets of the time series beginning at t = 0 days, and ending at t = *x* days. As per the above analysis, we fit cell shape

metrics to power law models or otherwise and used a F-test to determine the probability that the data could be from the same data set ($P > 0.05$). We then increased the time *x* until $P < 0.05$. The last time at which $P > 0.05$ is reported as the time at which cell shape is similar (as per Table 1).

**FFT analysis – processing**

To quantify the alignment of cells we compared two dimensional fast Fourier transforms (2D FFT) of images obtained from time-lapse microscopy. The power spectrum of the 2D FFT of an images can be used to compare the presence of features at a given orientation.[5] Summing the power spectrum of 2D FFT images at a given angle the prominence of cell features at given orientations is compared between the different substrate types. This method is advantages as it can provide orientation distributions for images of overlapping features. Further, its speed and robust nature make it the preferred calculation methods for quantification of alignment distributions in this large data set. Prior to calculation of 2DFFTs, the background of each image set was subtracted from each image. The background image was created from initially images prior to cell spreading and movement. Using MATLAB 2015b, a FFT transform of each frame of the fluorescent LifeAct-GFP channel of time-lapse microscopy was taken. The magnitude spectrum was rearranged to move zero frequency components to the center of the FFT array. The angle of each pixel from the center was calculated and its saturation summed to give the relative strength of different angle bands within the FFT array. Points close to the center of the array were excluded as their close location to the center corresponds to low angular resolution and results in data skewed to certain angles. This gave the total power of the 2D FFT at a given angle and could be used to compare the presence of features at a given orientation. For each topography type, the power spectrums from each field of view at a given time point were combined and normalized. Following calculation of the power spectrums across the complete 21 days, spectrums for a given substrate were combined and graphed as a surface (as per Figure 6 in main article).

**Representative videos of LifeAct GFP (actin) channel of cells over various topographies:**

random
https://drive.google.com/file/d/0ByVrOiB-UfWYVDJvZmRid1NDSTg/view?usp=sharing
flat
https://drive.google.com/file/d/0ByVrOiB-UfWYNWprdlVoUmJFM0k/view?usp=sharing
flat-osteo
https://drive.google.com/file/d/0ByVrOiB-UfWYclVfLUZjUmUxNlU/view?usp=sharing
align
https://drive.google.com/file/d/0ByVrOiB-UfWYNnBKN0ZHR29ESkU/view?usp=sharing

**References**

[1] M. Zhang, S. Fang, A. A. Zakhidov, S. B. Lee, A. E. Aliev, C. D. Williams, K. R. Atkinson, R. H. Baughman, *Science* **2005**, *309*, 1215.

[2] M. Zhang, *Science (80-. ).* **2005**, *309*, 1215.

[3] J. Riedl, A. H. Crevenna, K. Kessenbrock, J. H. Yu, D. Neukirchen, M. Bista, F. Bradke, D. Jenne, T. A. Holak, Z. Werb, M. Sixt, R. Wedlich-Soldner, *Nat. Methods* **2008**, *5*, 605.

[4] C. A. Reinhart-King, M. Dembo, D. A. Hammer, *Biophys. J.* **2005**, *89*, 676.

[5] W. M. Petroll, H. D. Cavanagh, P. Barry, P. Andrews, J. V Jester, *J. Cell Sci.* **1993**, *104 ( Pt* 2, 353.
